# Pilot study of myocardial ischemia-induced metabolomic changes in emergency department patients undergoing stress testing

**DOI:** 10.1371/journal.pone.0211762

**Published:** 2019-02-01

**Authors:** Alexander T. Limkakeng, Ricardo Henao, Deepak Voora, Thomas O’Connell, Michelle Griffin, Ephraim L. Tsalik, Svati Shah, Christopher W. Woods, Geoffrey S. Ginsburg

**Affiliations:** 1 Division of Emergency Medicine, Department of Surgery, Duke University, Durham, North Carolina, United States of America; 2 Center for Applied Genomics & Precision Medicine, Duke University, Durham, North Carolina, United States of America; 3 Division of Cardiology, Department of Medicine, Duke University, Durham, North Carolina, United States of America; 4 Indiana University, Indianapolis, Indiana, United States of America; 5 Emergency Medicine Service, Durham Veteran’s Affairs Medical Center, Durham, North Carolina, United States of America; 6 Division of Infectious Diseases & International Health, Department of Medicine, Duke University, Durham, North Carolina, United States of America; University of Palermo, ITALY

## Abstract

**Background:**

The heart is a metabolically active organ, and plasma acylcarnitines are associated with long-term risk for myocardial infarction. We hypothesized that myocardial ischemia from cardiac stress testing will produce dynamic changes in acylcarnitine and amino acid levels compared to levels seen in matched control patients with normal stress tests.

**Methods:**

We analyzed targeted metabolomic profiles in a pilot study of 20 case patients with inducible ischemia on stress testing from an existing prospectively collected repository of 357 consecutive patients presenting with symptoms of Acute Coronary Syndrome (ACS) in an Emergency Department (ED) observation unit between November 2012 and September 2014. We selected 20 controls matched on age, sex, and body-mass index (BMI). A peripheral blood sample was drawn <1 hour before stress testing and 2 hours after stress testing on each patient. We assayed 60 select acylcarnitines and amino acids by tandem mass spectrometry (MS/MS) using a Quattro Micro instrument (Waters Corporation, Milford, MA). Metabolite values were log transformed for skew. We then performed bivariable analysis for stress test outcome and both individual timepoint metabolite concentrations and stress-delta metabolite ratios (T2/T0). False discovery rates (FDR) were calculated for 60 metabolites while controlling for age, sex, and BMI. We built multivariable regularized linear models to predict stress test outcome from metabolomics data at times 0, 2 hours, and log ratio between these two. We used leave-one-out cross-validation to estimate the performance characteristics of the model.

**Results:**

Nine of our 20 case subjects were male. Cases’ average age was 55.8, with an average BMI 29.5. Bivariable analysis identified 5 metabolites associated with positive stress tests (FDR < 0.2): alanine, C14:1-OH, C16:1, C18:2, C20:4. The multivariable regularized linear models built on T0 and T2 had Area Under the ROC Curve (AUC-ROC) between 0.5 and 0.55, however, the log(T2/T0) model yielded 0.625 AUC, with 65% sensitivity and 60% specificity. The top metabolites selected by the model were: Ala, Arg, C12-OH/C10-DC, C14:1-OH, C16:1, C18:2, C18:1, C20:4 and C18:1-DC.

**Conclusions:**

Stress-delta metabolite analysis of patients undergoing stress testing is feasible. Future studies with a larger sample size are warranted.

## Introduction

Acute Coronary Syndrome (ACS) remains the leading cause of morbidity and mortality for Americans, accounting for 1 out of every 3 deaths [1]. Each year, over 6 million people are rushed to emergency departments (ED) with ACS symptoms and many more receive testing as outpatients [2]. Cardiac biomarkers and provocative stress testing are the mainstays of ACS risk assessment but have limitations [3–6].

Because the heart is a highly metabolically active organ, a variety of humoral metabolic assessments have been suggested to assist in risk stratification for ACS [7–9]. Metabolite factors such as medium and long chain acylcarnitines have been found to be associated with long-term risk for AMI or death in outpatient populations [10, 11]. Acylcarnitines are able to enter the mitochondria and are thus surrogate markers of fatty acid Beta-oxidation. As another example, angina patients have been demonstrated to have higher branched-chain amino acids and phenylalanine concentrations [12]. Sabatine et al. [13] compared a range of metabolic factors in 18 patients with inducible myocardial ischemia compared to matched controls who were undergoing stress testing, finding differential changes in 6 factors, including a number of amino acids. Studies of other forms of myocardial stress such as a planned ablation or bypass surgery indicate that metabolomic profiling can similarly identify early myocardial injury and ischemia [14, 15]. However, few studies have examined dynamic changes in metabolites and no prior studies have examined symptomatic patients undergoing stress testing in an emergency department.

Cardiac stress testing presents an ideal method to investigate early changes in the plasma associated with myocardial ischemia. Given that prior work has demonstrated that single-timepoint acylcarnitine and amino acid concentrations can risk stratify patients for subsequent cardiac events, we sought to demonstrate the feasibility of testing whether recently symptomatic ED patients with inducible myocardial ischemia on cardiac stress echocardiogram testing have distinct changes in blood acylcarnitine and amino acid levels compared to matched control patients who have normal stress tests. By obtaining multiple timepoints, we allow each patient to functionally act as their own control.

## Methods

### Study setting and population

We analyzed the baseline and 2-hour post-stress blood samples of 20 case patients with inducible ischemia on stress testing and 20 controls with normal stress tests who were matched for age, sex, and body-mass index (BMI). These patients were selected from a repository created from a prospective cohort study [16]. We briefly describe these methods here for reference. Samples were collected from ED patients at a single academic medical center with an approximate annual census of 75,000 visits. We recruited 357 patients from November 2012 to September 2014 from among consecutive patients presenting on weekdays (and intermittently on weekends) with symptoms suggestive of ACS. Subjects had ruled out for myocardial infarction by serial troponins and were assigned to an adjoining ED-based observation unit. Out of this repository, 27 patients had inducible ischemia on stress testing, from which our 20 cases were selected. Our sample size was thus limited by the number of patients with ischemia on stress testing.

Patients were older than 30 years of age and all underwent an exercise echocardiogram cardiac stress test testing. Prior to stress testing all patients had serial troponin assays performed (Roche Elecsys 4^th^ generation Troponin T (TnT) and were below institutional cutoff (<0.1 ng/mL) over 8 hours. We excluded patients with acute ischemic changes on ECG, significant arrhythmias, unstable vital signs, or other acute conditions that would preclude stress testing. All patients provided written informed consent to participate and the study protocol was approved by the Institutional Review Board of Duke University Medical Center.

We prospectively collected demographics and other relevant medical history, including laboratory testing, radiographic testing, and physical exam findings. We recorded any subsequent MI (ICD9 code from subsequent hospitalization); abnormal stress testing; significant coronary disease by angiography (lesions >50% in a major epicardial coronary artery), percutaneous coronary intervention, or coronary artery bypass graft surgery; death (cardiovascular and all-cause). Study data were collected and managed using REDCap (Research Electronic Data Capture) [17] electronic data capture tools hosted at Duke University.

### Sample collection and metabolomic analysis

A peripheral blood sample was drawn before stress testing, and at 2 hours after stress testing (2-hour Post-Stress Value). Blood was centrifuged within 1 hour for collection of plasma. This plasma was transferred into 0.5 mL aliquots and were frozen at -80°C within 8 hours of collection. Samples were drawn from existing intravenous catheters when possible.

Samples were processed at the Duke Molecular Physiology Institute. Given that prior work by one of the authors demonstrated the predictive value of acylcarnitines for acute coronary syndrome in outpatients, we utilized the same methods to study acylcarnitines in the current subjects. Mass spectrometry was used to assay select acylcarnitines and amino acids, as previously described in detail [18]. The complete list of metabolites that we assayed are shown in S1 Table. Liquid-handling steps were routinely performed on a Genesis RSP 150/4 robotic sample processor (Tecan AG, Maennedorf, Switzerland). Quantitative measurement of targeted analytes was achieved by spiking of plasma samples with cocktails of stable isotope-labeled standards specific to each assay module prior to sample extraction or other manipulations [18]. For the preparation of plasma acylcarnitines and amino acids, the proteins were first removed by precipitation with methanol. Supernatant was dried and then esterified with hot, acidic methanol (acylcarnitines) or n-butanol (amino acids). Acylcarnitines and amino acids were then analyzed by tandem mass spectrometry (MS/MS) using a Quattro Micro instrument (Waters Corporation, Milford, MA). The amino acid (AA) lower level of quantitation (LOQ) was 0.5 μM and the acylcarnitine (AC) LOQ was 0.015 μM.

### Exercise stress echocardiography

Patients underwent standard exercise testing on a treadmill with echocardiography evaluation. We used the Bruce protocol with mandatory termination for angina, ≥3 mm ST-segment depression, decrease of ≥20 mmHg in systolic blood pressure, serious arrhythmias, fatigue, or severe dyspnea. Inducible ischemia was defined as the occurrence of new segmental wall motion abnormalities during stress. The stress echocardiograms were interpreted as per usual care by board-certified cardiologists who were blinded to the results of study-specific metabolomic testing.

Two reviewers further reviewed stress test reports of all patients with abnormal tests. Disagreements or remaining indeterminate reports were adjudicated by a third reviewer, a board-certified cardiologist, using the entire stress test report and clinical records.

### Primary data analysis

Our goal was to determine the feasibility of this analysis and some of the parameters that would impact future study of this stress-delta metabolomics paradigm. We assessed bivariable associations between stress test outcome (case vs. control) and clinical variables. For continuous variables we used Student’s t test and for categorical variables Chi-square test. We also performed bivariable associations between stress test outcome and individual metabolite concentrations from pre- and post-test timepoint, and between outcomes and metabolite ratios (metabolite at T2/metabolite at T0) using generalized linear models. False discovery rates (FDR) were calculated for each of 60 metabolites. We controlled for clinical variables relevant to the outcome, namely, aspirin use, history of smoking, hypertension, and hyperlipidemia. The following confounded variables were not considered in the analyses: history of coronary artery disease, revascularization, and angiography.

We developed multivariable regularized linear models to predict stress test outcome from metabolomics data at times 0, 2 hours, and log ratio between these two. We used nested Leave-One-Out Cross-Validation (LOOCV) to estimate the performance characteristics of the model. LOOCV allows us to obtain an estimation of the performance metric (AUC-ROC) that is unbiased and independent of the estimation of the parameters (regression coefficients) and hyperparameters of the model (regularization coefficients). This is done by estimating the parameters and hyperparameters of the model on the N (sample size) subsets of size N-1, then estimating performance on the predictions made on the left-out samples (one on each iteration). Confidence intervals for AUC-ROC, sensitivity and specificity were estimated using bias corrected and accelerated percentile bootstrapping. For further exploratory analyses, we examined whether classification of cases as only those patients with angiographically proven coronary artery stenosis >50% or those patients requiring coronary artery bypass grafting (CABG) impacted results, or whether categorizing abnormal stress tests in ordinal fashion by severity of ischemia induced (as opposed to dichotomously) would change our analysis. Based on a prior study from this repository (submitted, under consideration), we also secondarily analyzed whether pro-B-type natriuretic peptide (pro-BNP) levels could distinguish cases from controls. All analyses were done in R core stats package [19].

For exploratory purposes, based on the effect sizes we observed, we performed a sample size calculation for a future study that would have 90% power to detect at alpha <0.05 an effect size d of 0.5 of delta-metabolite levels between groups using an unmatched t test. We calculated sample sizes required to study only one candidate of interest, or 5 metabolites with a Bonferroni correction.

## Results

Table 1 shows the demographic and clinical characteristics of chosen patients, and each individual’s characteristics are shown in S2 Table. The prevalence of known coronary artery disease and of coronary artery disease risk factors was low. Nine of our 20 case subjects were male. Cases’ median age was 55.5 and median BMI 30.1.

**Table 1:** Demographics and characteristics of enrolled patients.

Characteristic	Cases N (%)	Controls N (%)	p values
Age (Years), Median (Range)	55.5 (42–79)	55.0 (44–71)	0.876
Sex			0.999
Male	9 (45%)	9 (45%)	
Female	11 (55%)	11 (55%)	
Race			0.538
Asian	0 (0%)	1 (5%)	
Black or African American	7 (35%)	7 (35%)	
White / Caucasian	13 (65%)	12 (60%)	
Other	0 (0%)	1 (5%)	
Hypertension	14 (70%)	5 (25%)	0.004
Diabetes	5 (25%)	2 (10%)	0.21
History of Tobacco Use	13 (65%)	8 (40%)	0.11
Hyperlipidemia	13 (65%)	2 (10%)	0.0003
Past Myocardial Infarction	3 (15%)	1 (5%)	0.29
Coronary Artery Disease	6 (30%)	0 (0%)	0.008

Individual metabolite levels for each timepoint in each subject are shown in S3 Table and boxplots are shown in S1 and S2 Figs Among clinical variables tested, hypertension and hyperlipidemia were associated with stress test outcome (p < 0.05). Coronary artery disease, history of revascularization, and history of angiography were not used in the model due to confounding in cases.

**Fig 1:**
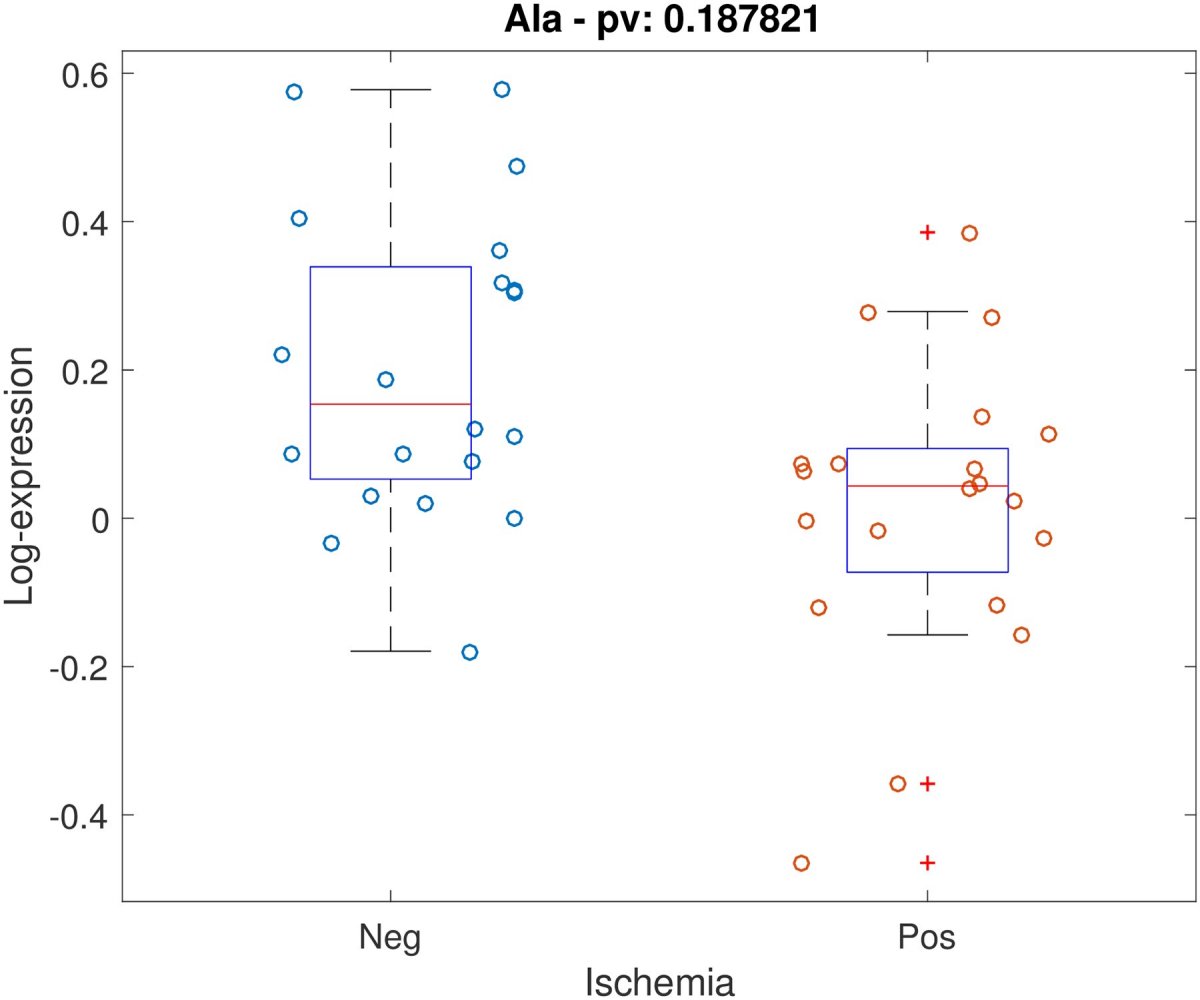
Scatter plot of stress-delta in alanine for patients who were positive versus negative for ischemia. When assessing changes in metabolite levels across timepoints while controlling for age, gender and BMI, 5 metabolites had FDR < 0.2: alanine, C14:1-OH, C16:1, C18:2, C20:4.

**Fig 2:**
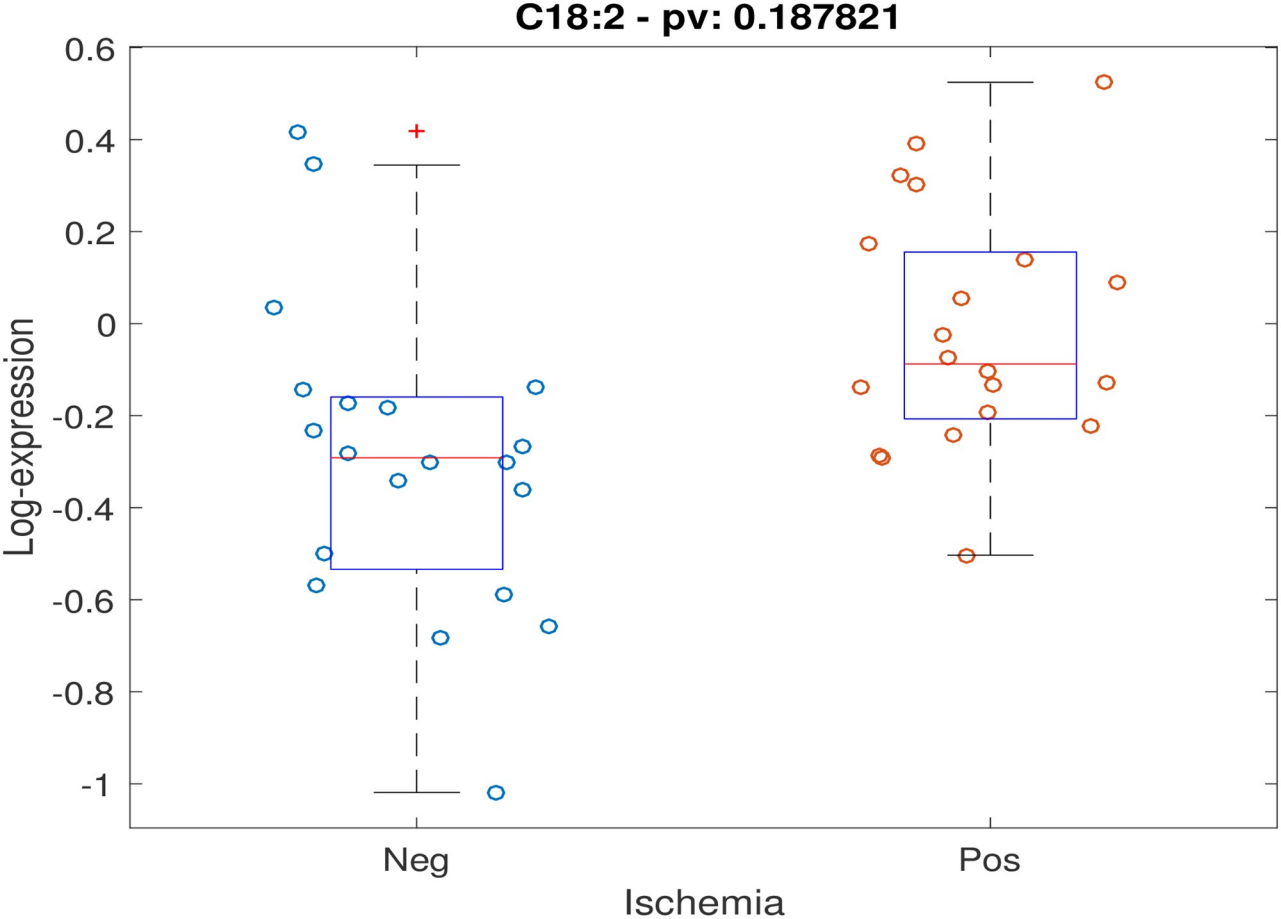
Scatter plot of stress-delta in C18:2 for patients who were positive versus negative for ischemia. When assessing changes in metabolite levels across timepoints while controlling for age, gender and BMI, 5 metabolites had FDR < 0.2: alanine, C14:1-OH, C16:1, C18:2, C20:4.

**Fig 3:**
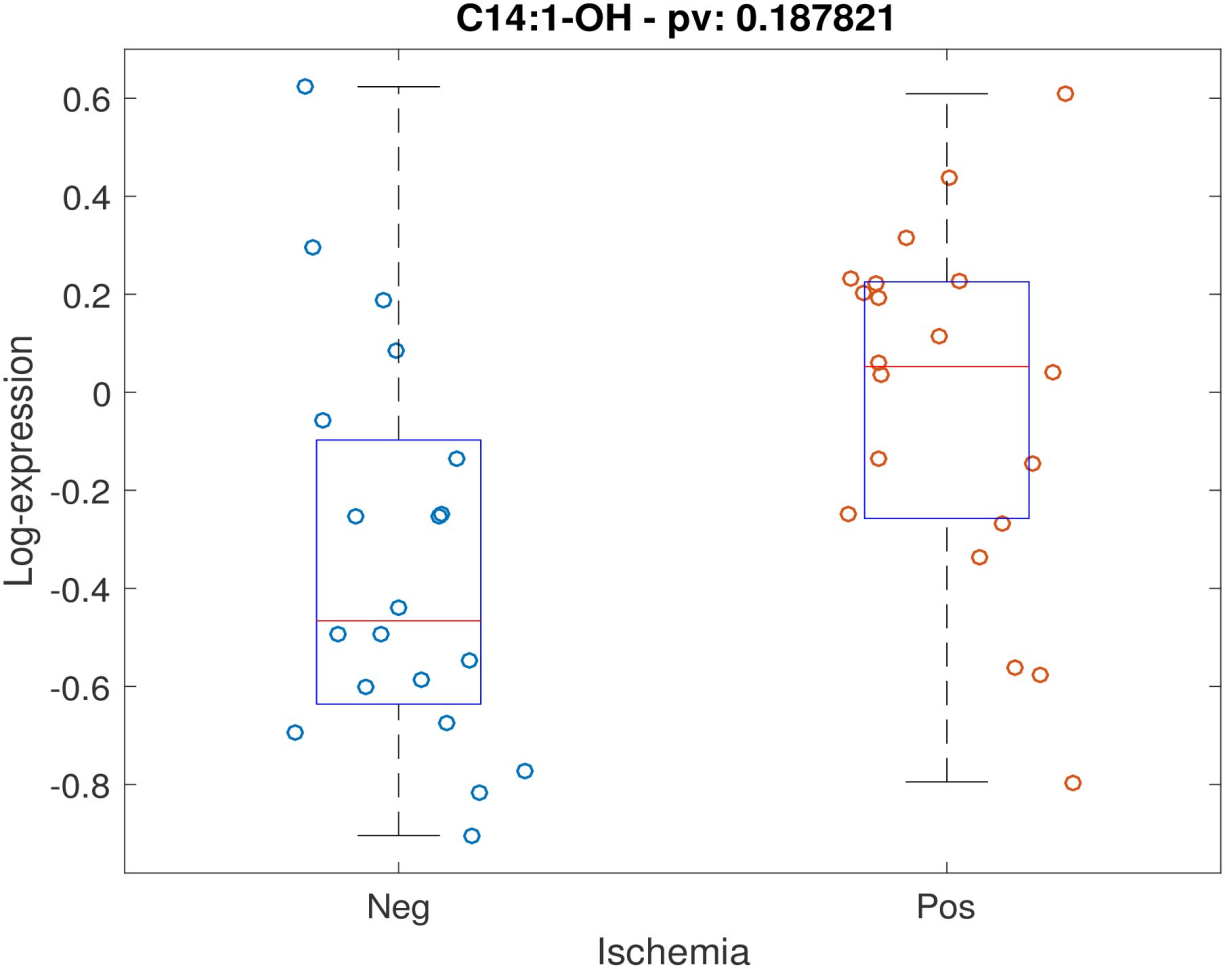
Scatter plot of stress-delta in C14:1-OH for patients who were positive versus negative for ischemia. When assessing changes in metabolite levels across timepoints while controlling for age, gender and BMI, 5 metabolites had FDR < 0.2: alanine, C14:1-OH, C16:1, C18:2, C20:4.

**Fig 4:**
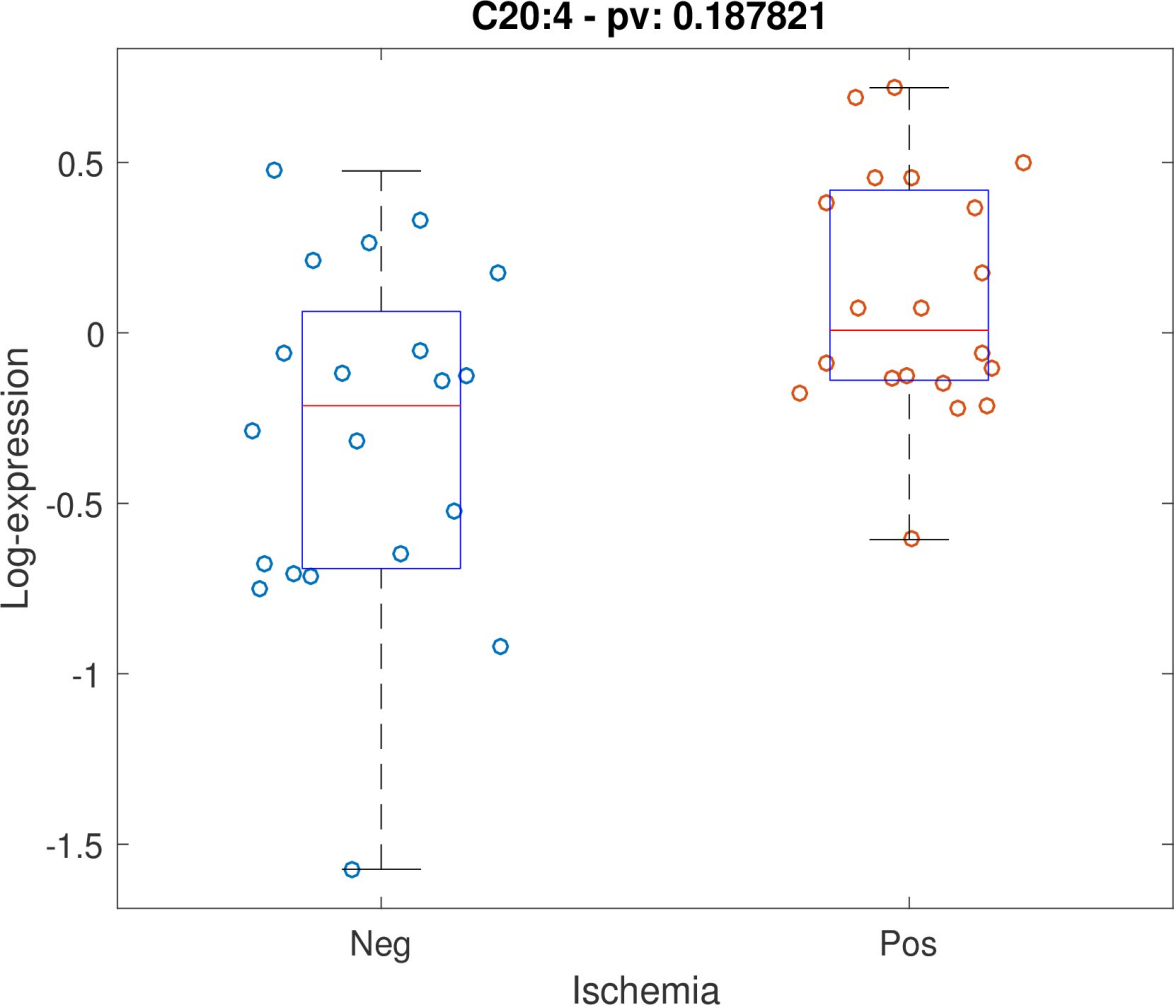
Scatter plot of stress-delta in C20:4 for patients who were positive versus negative for ischemia. When assessing changes in metabolite levels across timepoints while controlling for age, gender and BMI, 5 metabolites had FDR < 0.2: alanine, C14:1-OH, C16:1, C18:2, C20:4.

**Fig 5:**
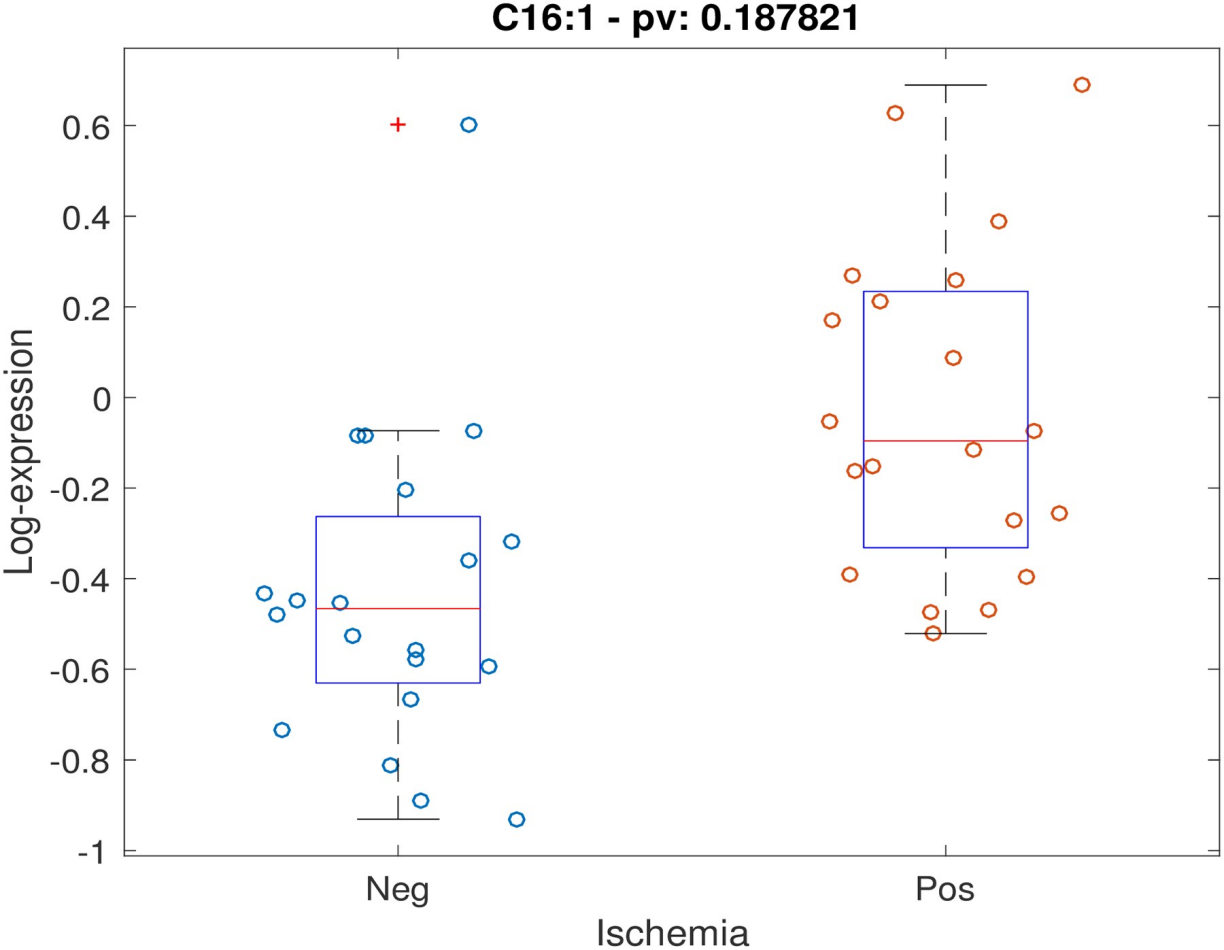
Scatter plot of stress-delta in C16:1 for patients who were positive versus negative for ischemia. When assessing changes in metabolite levels across timepoints while controlling for age, gender and BMI, 5 metabolites had FDR < 0.2: alanine, C14:1-OH, C16:1, C18:2, C20:4.

**Fig 6:**
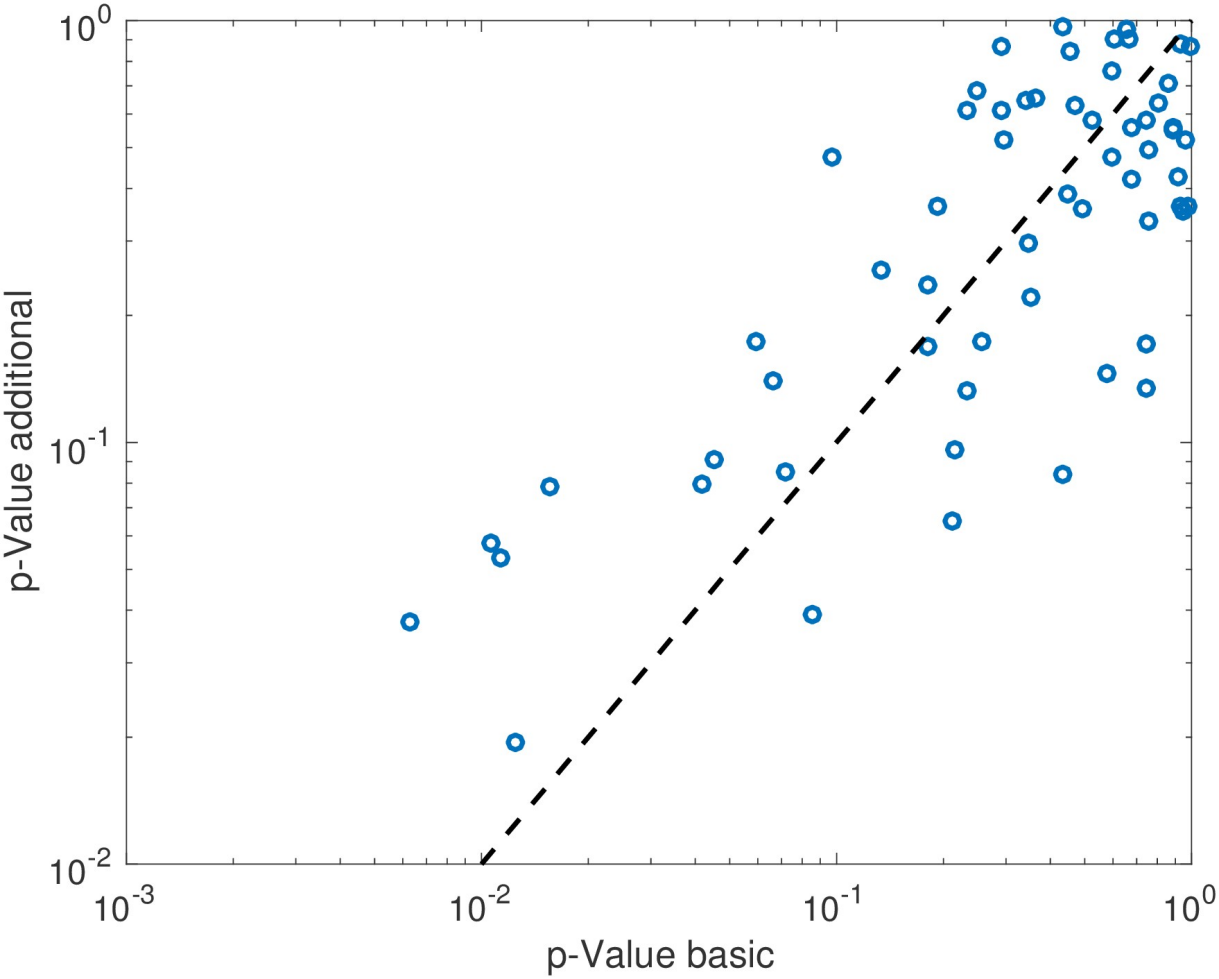
Raw p-values of metabolites after controlling for clinical variables. After controlling for aspirin, history of smoking, hypertension and hyperlipidemia, raw p-values correlated.

Positive stress test was not associated with individual timepoint metabolite concentrations at either timepoint with FDR < 0.2. Testing for association between stress test outcome and metabolite timepoint ratios (changes in levels across timepoints) while controlling for age, gender and BMI, demonstrated 5 metabolites with FDR < 0.2: alanine, C14:1-OH, C16:1, C18:2, C20:4. Scatter plots for these 5 metabolites are shown (Figs 1–5). After controlling for aspirin, history of smoking, hypertension, and hyperlipidemia, there were no metabolites with FDR < 0.2. However, raw p-values were still correlated (Fig 6).

The multivariable regularized linear models built on T0 and T2 had Area Under the ROC Curve (AUC-ROC) values between 0.5 and 0.55, however, the log(T2/T0) model yielded 0.625 (IQR 0.558 0.682) AUC, with 65% (IQR 0.570 0.720) sensitivity and 60% (IQR 0.667 0.524) specificity. The top metabolites selected by the model were: Alanine, Arginine, C12-OH/C10-DC, C14:1-OH, C16:1, C18:2, C18:1, C20:4 and C18:1-DC. Coefficients of the model and associated statistics are shown in Table 2.

**Table 2:** Multivariable regularized linear model coefficients.

Metabolite	Coefficient	Standard Error	*t* Statistic	*p*-value
**Alanine**	-3.708	4.218	-0.879	0.379
**Arginine**	-8.131	6.001	-1.355	0.175
**C12-OH/C10-DC**	3.461	2.090	1.656	0.098
**C14:1-OH**	-1.904	2.267	-0.840	0.401
**C16:1**	9.018	4.252	2.121	0.034
**C18:2**	6.445	4.032	1.599	0.110
**C18:1**	-11.738	5.793	-2.026	0.043
**C20:4**	2.181	1.936	1.127	0.260
**C18:1-DC**	-4.299	2.412	-1.782	0.075

In our further exploratory analyses, defining cases as only those patients with angiographically proven stenosis or CABG did not directionally change comparisons of metabolite levels or changes in metabolite levels. When we considered categorizing stress tests ordinally, we found that it impacted only 5 patients’ categorization and thus did not impact results. Finally, an analysis of pro-BNP levels demonstrated significant differences between baseline and 2-hour levels but not the change in levels based on stress test results. A similar analysis of pro-BNP using angiographically proven stenosis or CABG as the cases demonstrated no significant differences between groups in pro-BNP levels. Furthermore, our sample size calculation for a future study demonstrated that for a future study analyzing one metabolite, the largest sample size required (based on the differences observed for C20:4) would be 178 total patients. If a future study analyzed 5 metabolites of interest, using Bonferroni correction for 5 metabolites, a total of 220 patients would be required.

## Discussion

There is considerable interest in blood-borne markers of myocardial ischemia and early myocardial injury. Metabolomic profiles have been proposed as one approach for identifying these entities, both through systems biology approaches and as a means to identify traditional biomarker candidates. In this study, we matched patients with ischemia on exercise stress testing to patients without ischemia to compare metabolomic profile changes from baseline to 2 hours after their stress test. We have preliminarily found 5 metabolites that appear to change in concentration differently in patients with myocardial ischemia compared to those without on standard stress testing. We consider this to be hypothesis-generating work for further exploration and validation, but demonstrates the feasibility of the data collection and statistical analysis.

Others have attempted to identify metabolomic signatures of early myocardial injury with different models. In patients undergoing a planned myocardial infarction for treatment of hypertrophic cardiomyopathy, a metabolic signature consisting of aconitic acid, hypoxanthine, trimethylamine N-oxide, and threonine differentiated patients with myocardial injury from those undergoing diagnostic coronary angiography [14, 20]. Turer et al. studied patients undergoing cardiac surgery and found that the patient’s baseline ventricular health predicted metabolic responses to reperfusion and ischemia [15]. In their study, 2 specific acylcarnitine species were released following reperfusion: acetylcarnitine and 3-hydroxybutyryl-carnitine. They further found global limit of uptake of metabolic fuel in patients with underlying dysfunctional ventricles.

Numerous efforts have previously examined systems biology approaches to differentiating ST elevation myocardial infarction (STEMI) patients from normal controls. Some have used untargeted approaches to determine a metabolomic signature or transcriptomic approaches to differentiate STEMI from unstable angina or healthy controls [21, 22]. Previous investigators have demonstrated significant metabolic dysfunction in fatty acid metabolism in patients with coronary artery stenosis [23]. DeFilippis et al. demonstrated 19 metabolites with an intra-subject fold change from time of acute thrombotic MI presentation to the quiescent state, including lipids 2-hydroxybutyrate, and amino acids [24]. In other analyses of STEMI patients, lysophosphatidylcholines, caffeine, glycolysis, tryptophan and sphingomyelin metabolism and minerals were found to be disrupted compared to healthy controls [25]. We were interested in determining whether myocardial ischemia, which occurs earlier in the infarction process, could be detected through metabolomic signatures.

Prior work examining metabolic profiles in patients undergoing exercise generally found that exercise-induced increases in glycerol were strongly related to fitness levels in normal individuals and were attenuated in subjects with myocardial ischemia [26]. Sabatine et al. published the earliest report on metabolomic analysis of patients undergoing cardiac stress testing [13]. They identified several amino acids that allowed differentiation of patients with ischemia on their stress test. In this study, we add deeper analysis of acylcarnitines to this model and examined recently symptomatic patients in our emergency department observation unit.

Our work, though preliminary, is in alignment with other research that indicates resting metabolomic profiles can discriminate over and above traditional ACS risk stratification systems, even in asymptomatic patients [11, 27]. In particular, medium and long chain acylcarnitines have been associated with increased risk. This may reflect an underlying defect in mitochondrial function and Beta-oxidation, which have been shown to be a mechanism underlying increased insulin resistance [18, 28]. These processes may also increase inflammation within the cardiovascular system, leading to increased likelihood of atherosclerotic plaque rupture.

Based on this prior work, we studied the same metabolites in our patients undergoing provocative cardiac stress testing to determine whether the changes identified in the prior studies occur early in the ischemia process. Prior work has examined patients with acute irreversible infarction and/or patients undergoing elective coronary intervention [21]. They likewise discovered that palmitic acid, linoleic acid, stearic acid and oleic acid, (all 16 or 18-carbon chain fatty acids) were relatively elevated in the serum of STEMI patients. This further confirms that fatty acid oxidation, as evidenced by elevated levels of acylcarnitines, is dysfunctional in the ischemic myocardial cell.

We propose that one reason such work was not able to identify differences between healthy control and elective coronary intervention (“unstable angina”) patients may be that the unstable angina patients’ blood samples were obtained long after their symptomatic episodes. In our model, we examined two timepoints in close proximity to a cardiac stress test as opposed to single resting baseline measurements. This allowed us to look for dynamic changes that occur early in the ischemia process.

There may be serum metabolite changes that are identifiable early in the process of acute myocardial ischemia. Further investigation into the metabolomic changes that occur early in the ischemia process may shed light on the changes that can be expected over longer periods of time. If so, this would not only inform our understanding of myocardial ischemia, but suggest an alternative clinical method for detecting inducible ischemia. Such work may produce new biomarkers of myocardial ischemia and/or panels of markers that can identify patients at short and long-term risk of adverse cardiac events. Our sample size calculations indicate that only a modest sized study of 220 patients would be required to validate these findings under the current conditions of age, sex, and BMI-matched cases and controls. A larger validation study that does not match cases with controls might require a larger study sample, since there would likely be larger variation between non-matched group comparisons.

### Limitations

This study is limited by small sample size. While we are underpowered to make definitive statements about the changes observed in metabolites following ischemia on stress testing, we conducted this pilot to prove feasibility of obtaining multiple samples before and after stress testing and assaying and analyzing multiple metabolites. A further limitation is that samples were acquired 2 hours following stress testing, which may be too late after an event to detect some metabolic changes. This timing was dictated by the repository’s parent study protocol. Patients were classified as either case or control on the basis of standard of care stress echocardiography readings. There is some variation in intra-observer agreement for this test. Because patients were enrolled from an observational registry, we were unable to control for workup bias downstream from the stress test. Therefore, some case patients had confirmatory cardiac angiography whereas others did not. Furthermore, even among those patients who are found to have significant coronary artery stenosis, there is some question over how important of a patient-oriented outcome this represents.

## Conclusion

We studied whether there were dynamic changes in a large number of selected metabolites between patients with myocardial ischemia on stress testing compared to matched normal controls, preliminarily identifying 5 metabolites that appear to change differentially. It is unclear whether these results can contribute additional information above standard clinical evaluation for myocardial ischemia. Delta-metabolite concentrations may offer more discriminative value than static single-timepoint metabolite measurement. This model of metabolomic exploration warrants further investigation.

## Supporting information

S1 Table List of analyzed metabolites. Sixty amino acids and acylcarnitine concentrations were measured in each plasma sample.
(XLSX)

S2 Table Detailed patient characteristics. Each patient’s individual demographic and clinical history data are shown.
(XLSX)

S3 Table Individual metabolite level results. Individual metabolite levels for each timepoint in each subject are shown. All units are μM. T0 = baseline and T2 = 2 hours after stress test.
(XLSB)

S1 Fig Acylcarnitine level boxplots in cases versus controls at both baseline and 2 hours after stress testing. Boxplots show mean, standard deviation, and range, with outliers. Pre = baseline levels. Post = 2 hours post-stress testing. All units are μM. Con = controls.
(PDF)

S2 Fig Amino acid level boxplots in cases versus controls at both baseline and 2 hours after stress testing. Pre = baseline levels. Post = 2 hours post-stress testing. All units are μM. Con = controls.
(PDF)
