# Supplementary material for: Pilot study of myocardial ischemia-induced metabolomic changes in emergency department patients undergoing stress testing
Source: PLoS One. 2019 Feb 1;14(2):e0211762. doi: 10.1371/journal.pone.0211762 (PMC6358091; doi:10.1371/journal.pone.0211762)
Supplement: S1 Fig — Boxplots show mean, standard deviation, and range, with outliers. Pre = baseline levels. Post = 2 hours post-stress testing. All units are μM. Con = controls. (PDF) [file pone.0211762.s004.pdf]

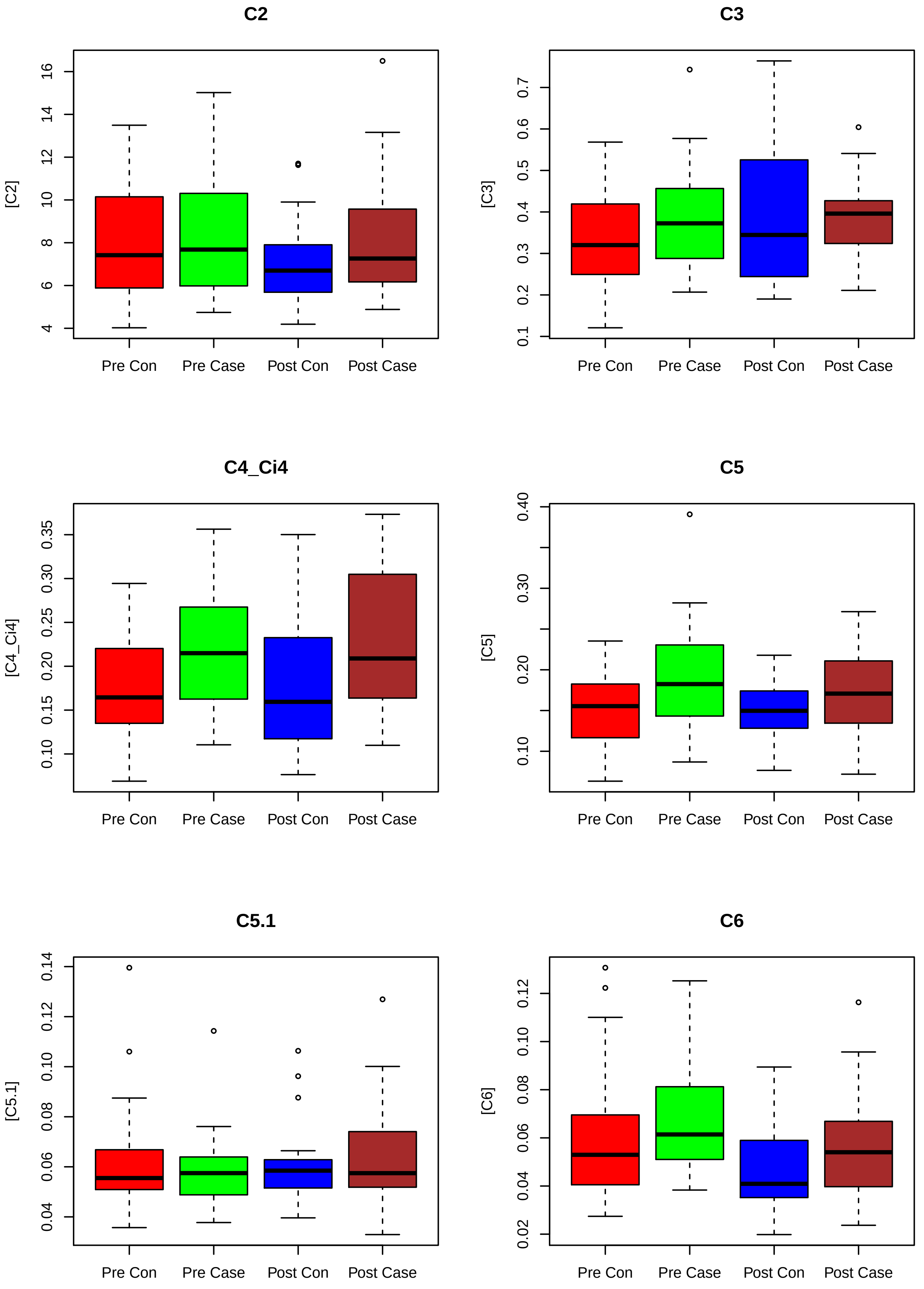
C2
[C2]
4
6
8
10
12
14
16
Pre Con
Pre Case
Post Con
Post Case
C3
[C3]
0.1
0.2
0.3
0.4
0.5
0.6
0.7
Pre Con
Pre Case
Post Con
Post Case
C4_Ci4
[C4_Ci4]
0.10
0.15
0.20
0.25
0.30
0.35
Pre Con
Pre Case
Post Con
Post Case
C5
[C5]
0.10
0.20
0.30
0.40
Pre Con
Pre Case
Post Con
Post Case
C5.1
[C5.1]
0.04
0.06
0.08
0.10
0.12
0.14
Pre Con
Pre Case
Post Con
Post Case
C6
[C6]
0.02
0.04
0.06
0.08
0.10
0.12
Pre Con
Pre Case
Post Con
Post Case

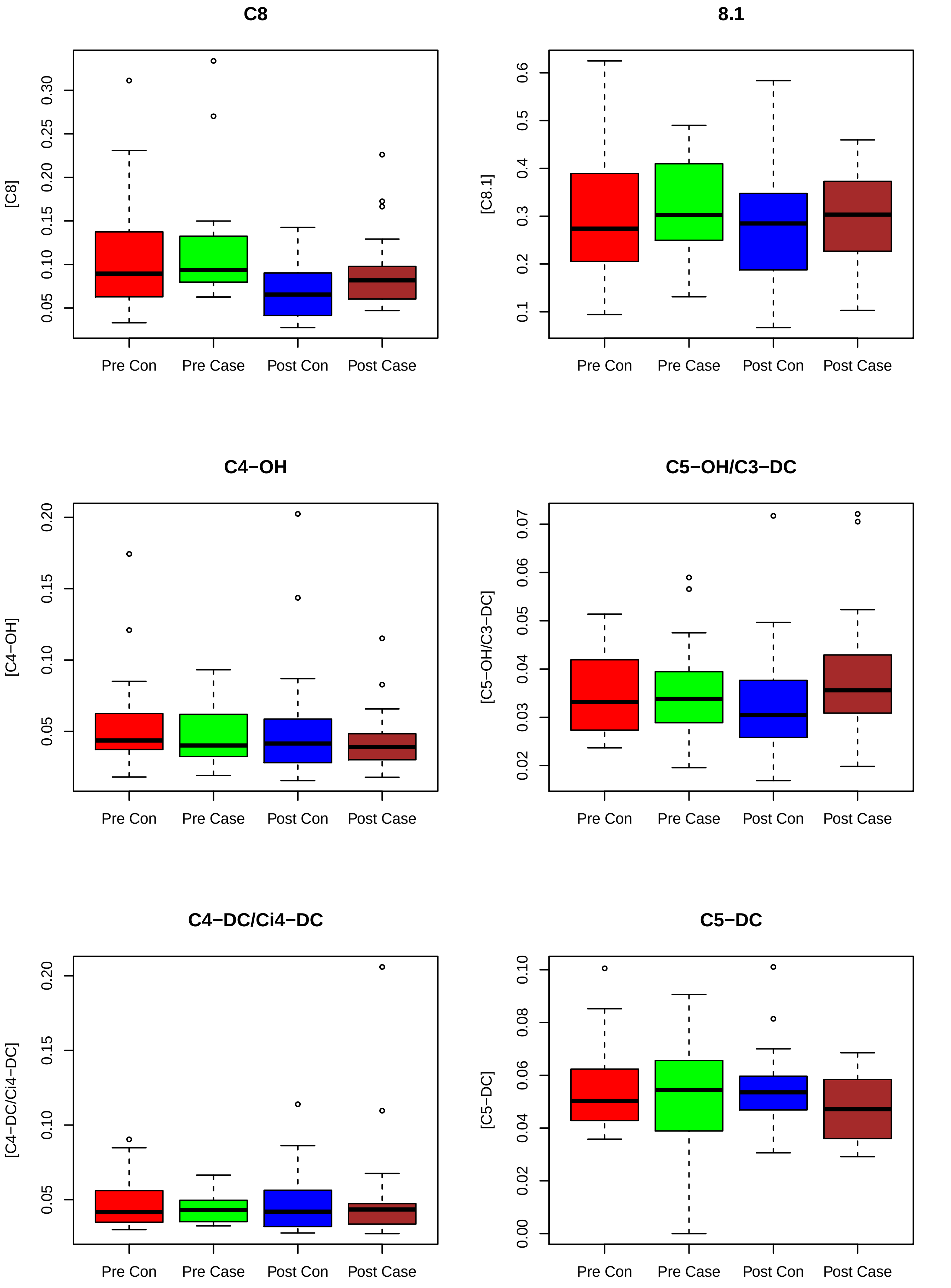
C8
[C8]
0.05
0.10
0.15
0.20
0.25
0.30
Pre Con
Pre Case
Post Con
Post Case
8.1
[C8.1]
0.1
0.2
0.3
0.4
0.5
0.6
Pre Con
Pre Case
Post Con
Post Case
C4−OH
[C4−OH]
0.05
0.10
0.15
0.20
Pre Con
Pre Case
Post Con
Post Case
C5−OH/C3−DC
[C5−OH/C3−DC]
0.02
0.03
0.04
0.05
0.06
0.07
Pre Con
Pre Case
Post Con
Post Case
C4−DC/Ci4−DC
[C4−DC/Ci4−DC]
0.05
0.10
0.15
0.20
Pre Con
Pre Case
Post Con
Post Case
C5−DC
[C5−DC]
0.00
0.02
0.04
0.06
0.08
0.10
Pre Con
Pre Case
Post Con
Post Case

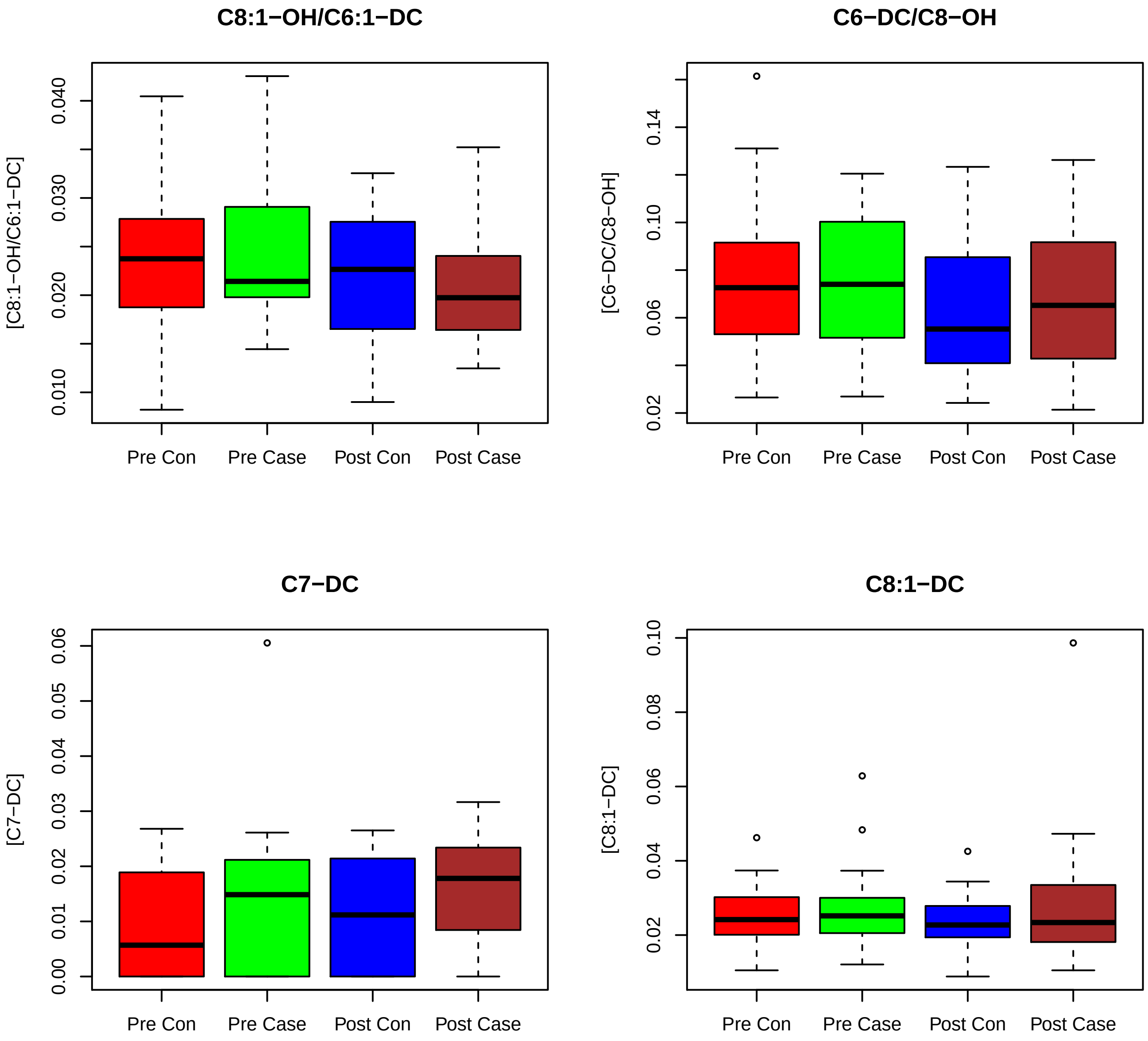
C8:1−OH/C6:1−DC
[C8:1−OH/C6:1−DC]
0.010
0.020
0.030
0.040
Pre Con
Pre Case
Post Con
Post Case
C6−DC/C8−OH
[C6−DC/C8−OH]
0.02
0.06
0.10
0.14
Pre Con
Pre Case
Post Con
Post Case
C7−DC
[C7−DC]
0.00
0.01
0.02
0.03
0.04
0.05
0.06
Pre Con
Pre Case
Post Con
Post Case
C8:1−DC
[C8:1−DC]
0.02
0.04
0.06
0.08
0.10
Pre Con
Pre Case
Post Con
Post Case

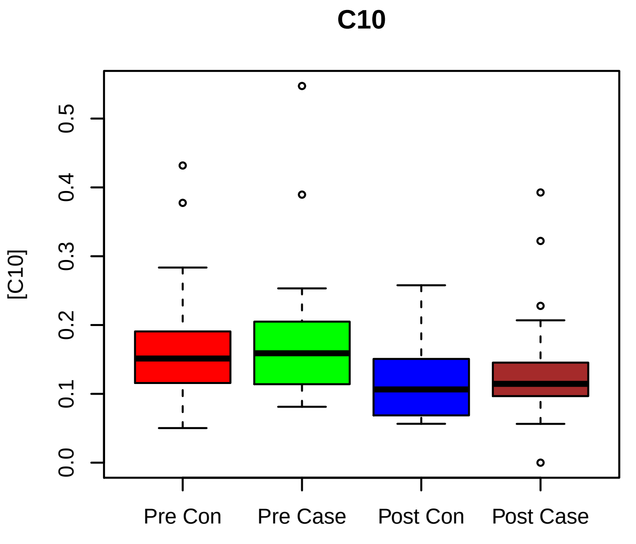
C10
[C10]
0.0
0.1
0.2
0.3
0.4
0.5
Pre Con
Pre Case
Post Con
Post Case

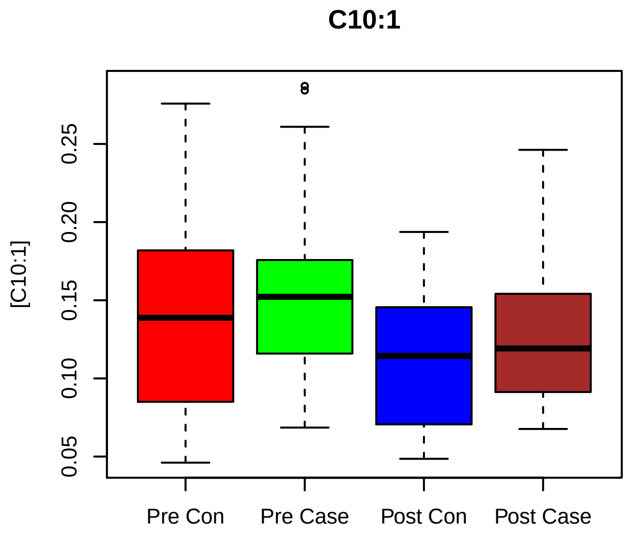
C10:1
[C10:1]
0.05
0.10
0.15
0.20
0.25
Pre Con
Pre Case
Post Con
Post Case

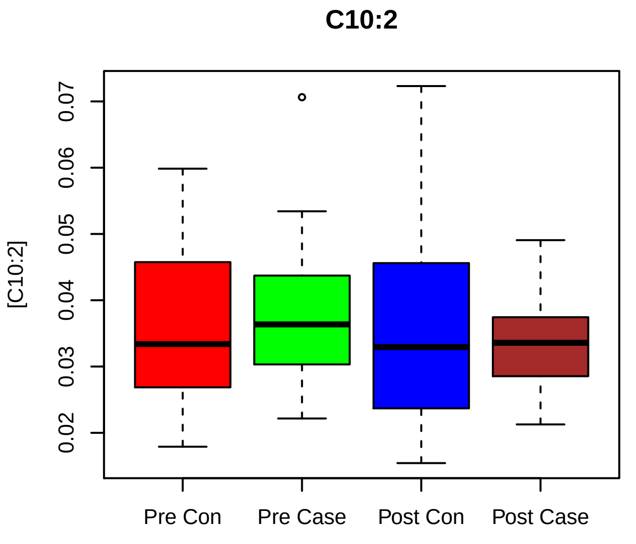
C10:2
[C10:2]
0.02
0.03
0.04
0.05
0.06
0.07
Pre Con
Pre Case
Post Con
Post Case

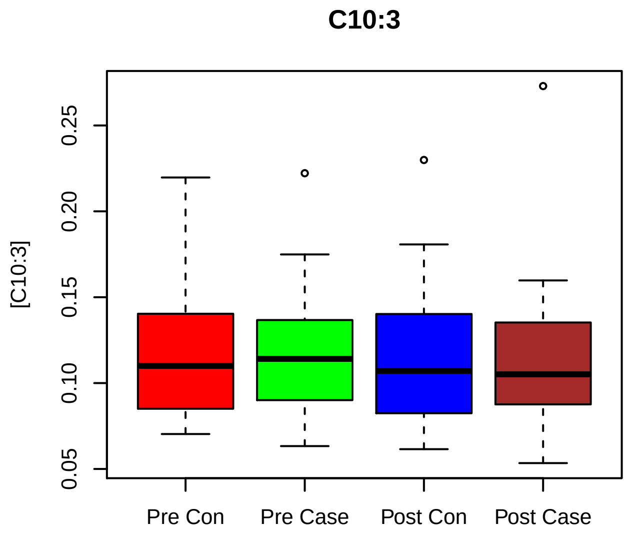
C10:3
[C10:3]
0.05
0.10
0.15
0.20
0.25
Pre Con
Pre Case
Post Con
Post Case

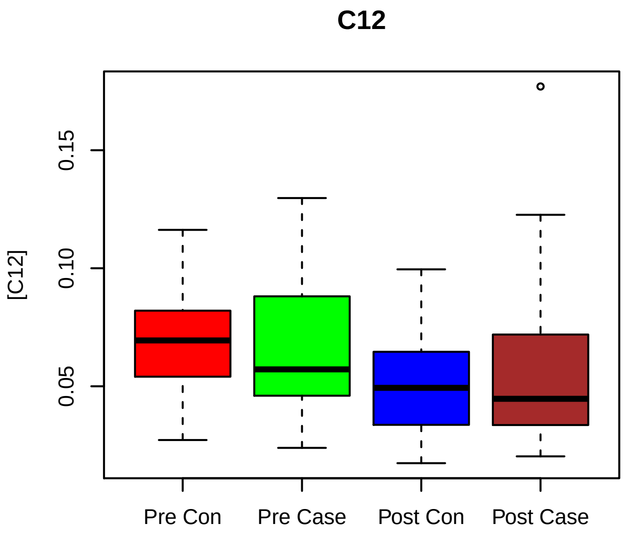
C12
[C12]
0.05
0.10
0.15
Pre Con
Pre Case
Post Con
Post Case

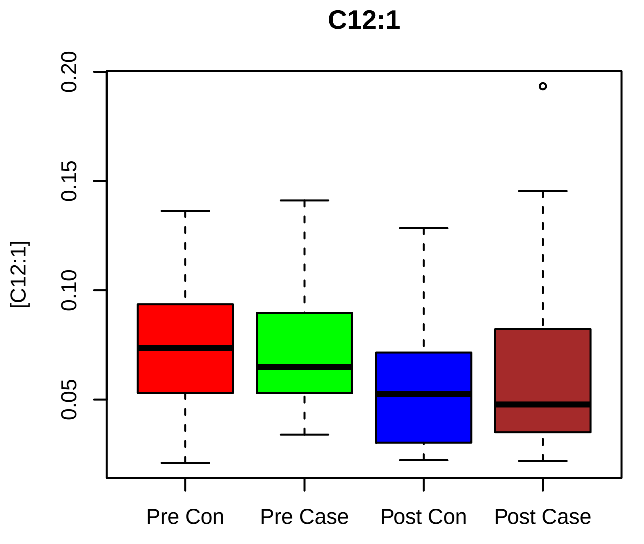
C12:1
[C12:1]
0.05
0.10
0.15
0.20
Pre Con
Pre Case
Post Con
Post Case

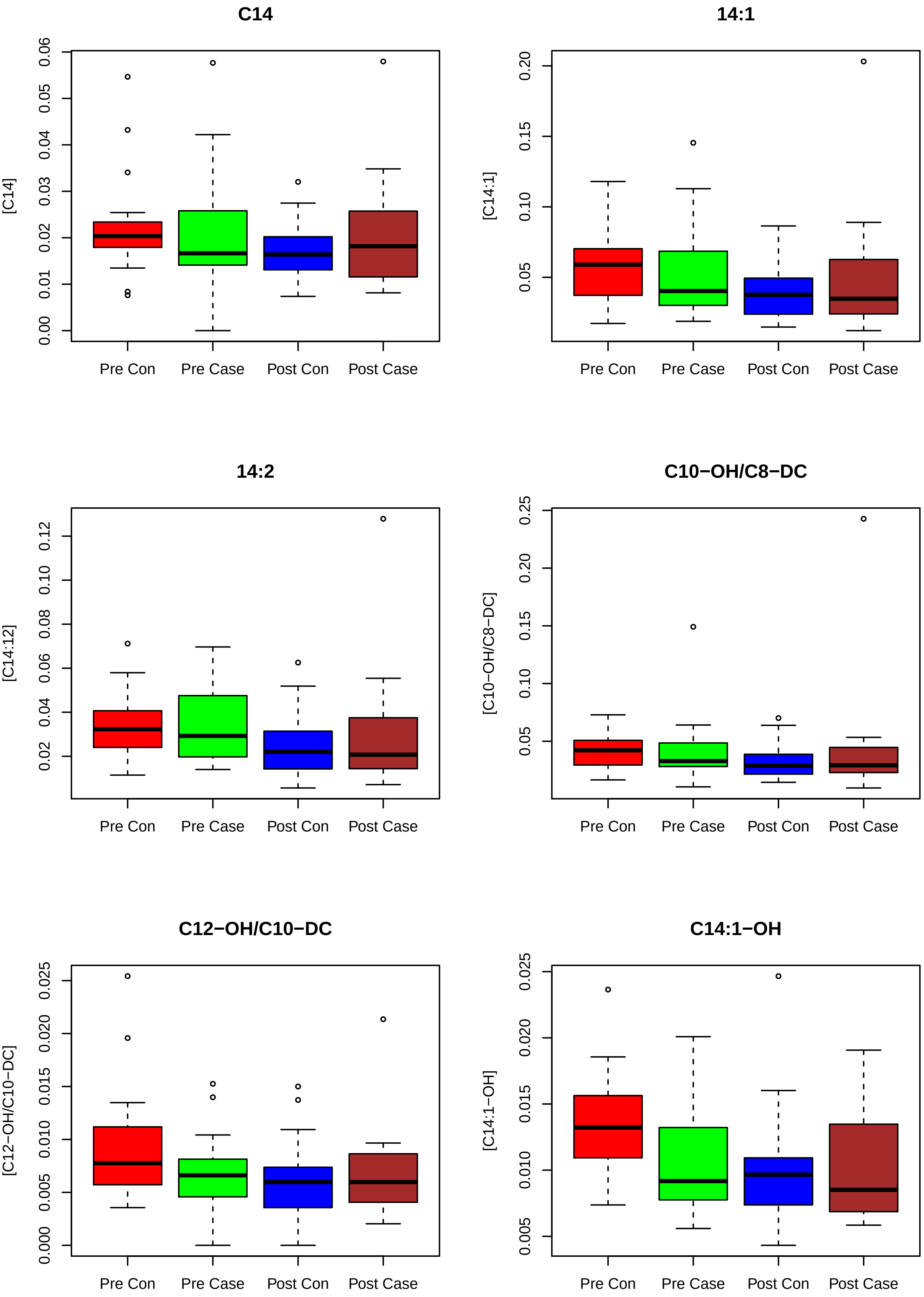
C14
[C14]
0.00
0.01
0.02
0.03
0.04
0.05
0.06
Pre Con
Pre Case
Post Con
Post Case
14:1
[C14:1]
0.05
0.10
0.15
0.20
Pre Con
Pre Case
Post Con
Post Case
14:2
[C14:12]
0.02
0.04
0.06
0.08
0.10
0.12
Pre Con
Pre Case
Post Con
Post Case
C10−OH/C8−DC
[C10−OH/C8−DC]
0.05
0.10
0.15
0.20
0.25
Pre Con
Pre Case
Post Con
Post Case
C12−OH/C10−DC
[C12−OH/C10−DC]
0.000
0.005
0.010
0.015
0.020
0.025
Pre Con
Pre Case
Post Con
Post Case
C14:1−OH
[C14:1−OH]
0.005
0.010
0.015
0.020
0.025
Pre Con
Pre Case
Post Con
Post Case

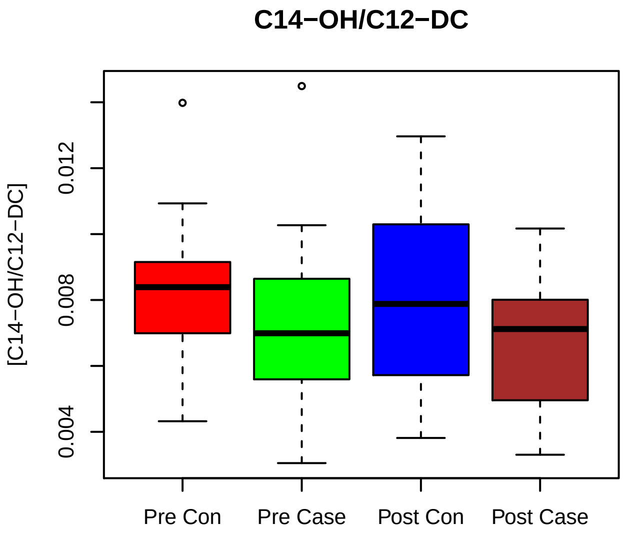
C14−OH/C12−DC
[C14−OH/C12−DC]
0.004
0.008
0.012
Pre Con
Pre Case
Post Con
Post Case

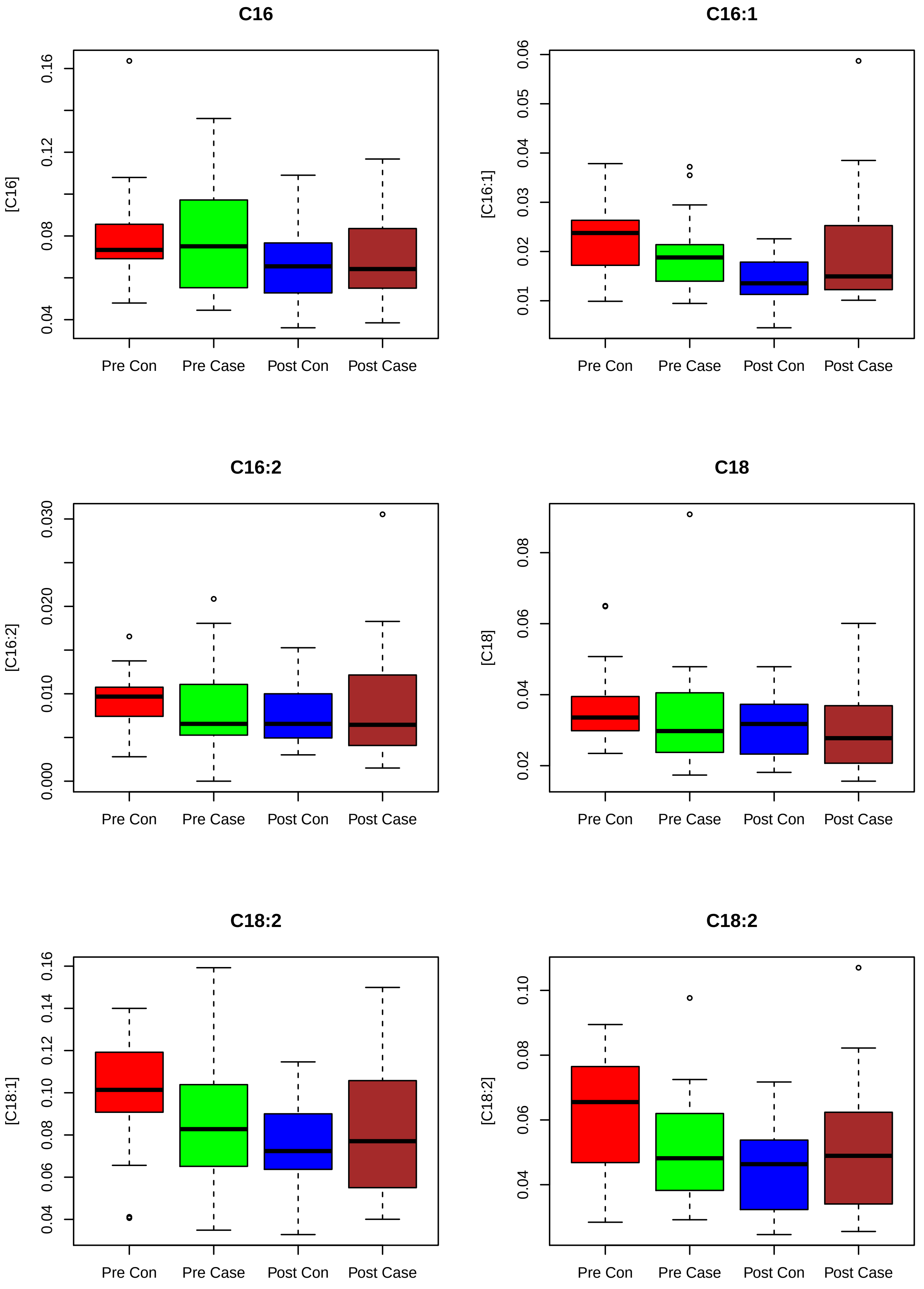
C16
[C16]
0.04
0.08
0.12
0.16
Pre Con
Pre Case
Post Con
Post Case
C16:1
[C16:1]
0.01
0.02
0.03
0.04
0.05
0.06
Pre Con
Pre Case
Post Con
Post Case
C16:2
[C16:2]
0.000
0.010
0.020
0.030
Pre Con
Pre Case
Post Con
Post Case
C18
[C18]
0.02
0.04
0.06
0.08
Pre Con
Pre Case
Post Con
Post Case
C18:2
[C18:1]
0.04
0.06
0.08
0.10
0.12
0.14
0.16
Pre Con
Pre Case
Post Con
Post Case
C18:2
[C18:2]
0.04
0.06
0.08
0.10
Pre Con
Pre Case
Post Con
Post Case

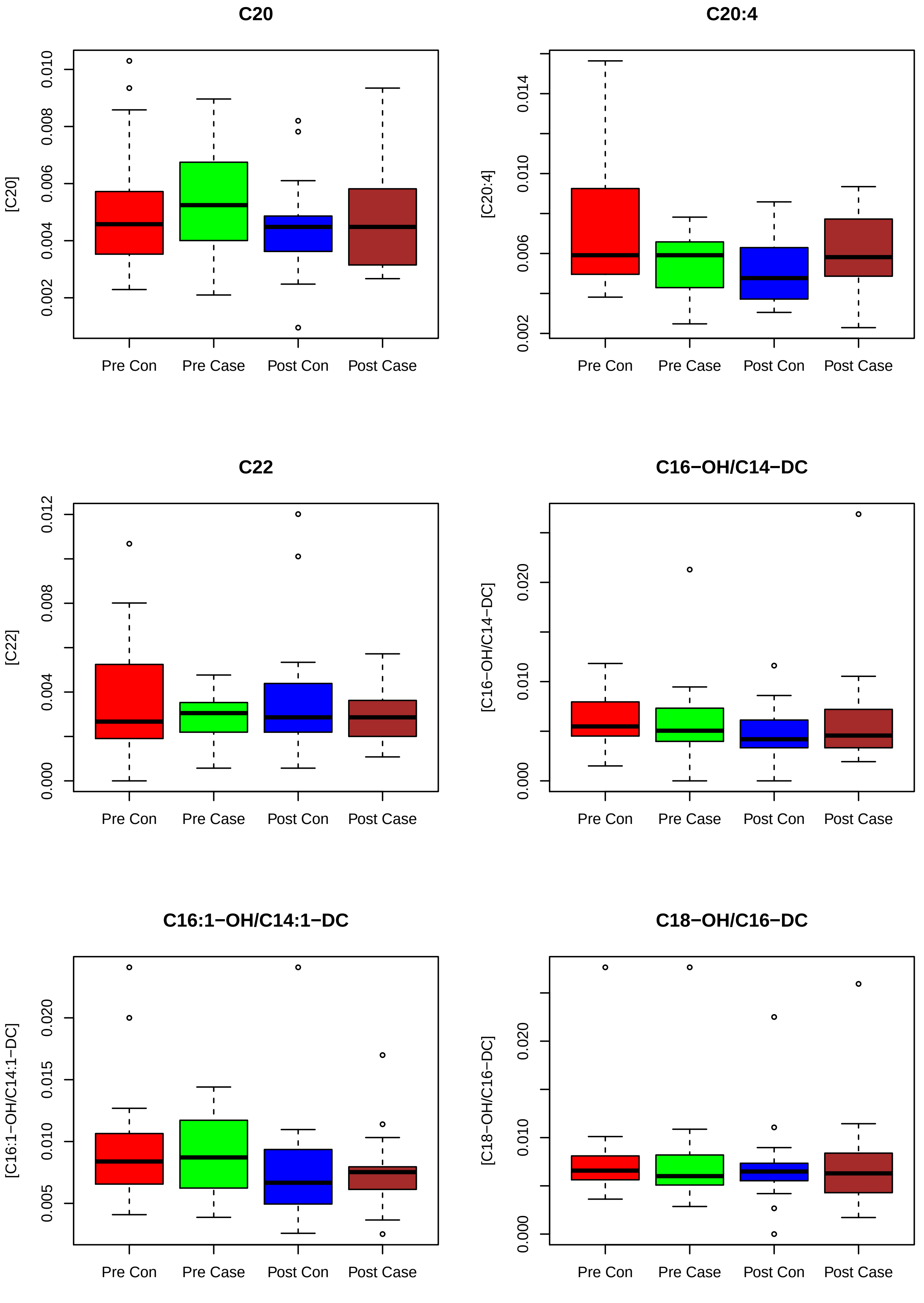
C20
[C20]
0.002
0.004
0.006
0.008
0.010
Pre Con
Pre Case
Post Con
Post Case
C20:4
[C20:4]
0.002
0.006
0.010
0.014
C22
[C22]
0.000
0.004
0.008
0.012
C16−OH/C14−DC
[C16−OH/C14−DC]
0.000
0.010
0.020
C16:1−OH/C14:1−DC
[C16:1−OH/C14:1−DC]
0.005
0.010
0.015
0.020
C18−OH/C16−DC
[C18−OH/C16−DC]
0.000
0.010
0.020

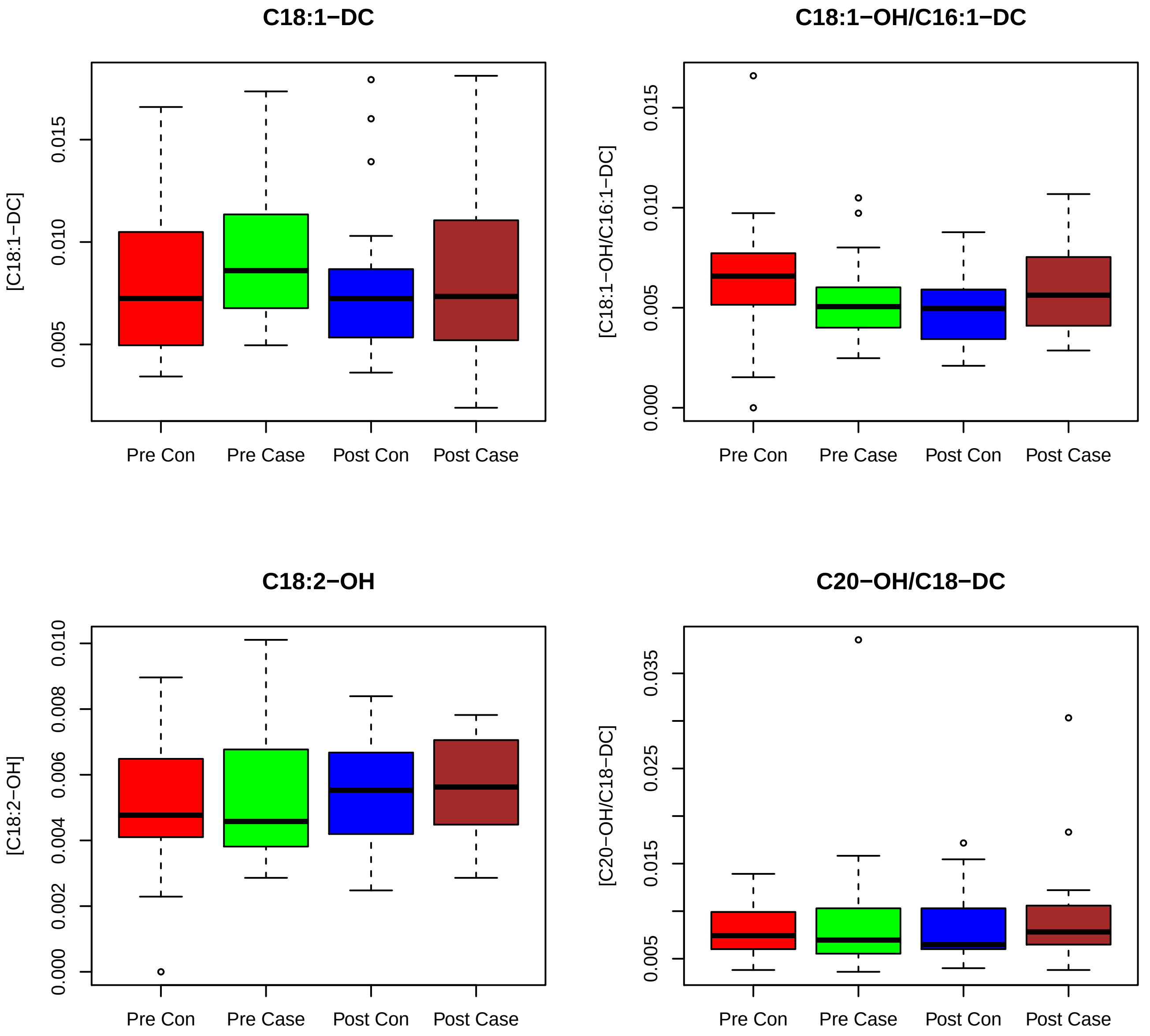
C18:1−DC
[C18:1−DC]
0.005
0.010
0.015
Pre Con
Pre Case
Post Con
Post Case
C18:1−OH/C16:1−DC
[C18:1−OH/C16:1−DC]
0.000
0.005
0.010
0.015
Pre Con
Pre Case
Post Con
Post Case
C18:2−OH
[C18:2−OH]
0.000
0.002
0.004
0.006
0.008
0.010
Pre Con
Pre Case
Post Con
Post Case
C20−OH/C18−DC
[C20−OH/C18−DC]
0.005
0.015
0.025
0.035
Pre Con
Pre Case
Post Con
Post Case
