# Supplementary material for: Pilot study of myocardial ischemia-induced metabolomic changes in emergency department patients undergoing stress testing
Source: PLoS One. 2019 Feb 1;14(2):e0211762. doi: 10.1371/journal.pone.0211762 (PMC6358091; doi:10.1371/journal.pone.0211762)
Supplement: S2 Fig — Pre = baseline levels. Post = 2 hours post-stress testing. All units are μM. Con = controls. (PDF) [file pone.0211762.s005.pdf]

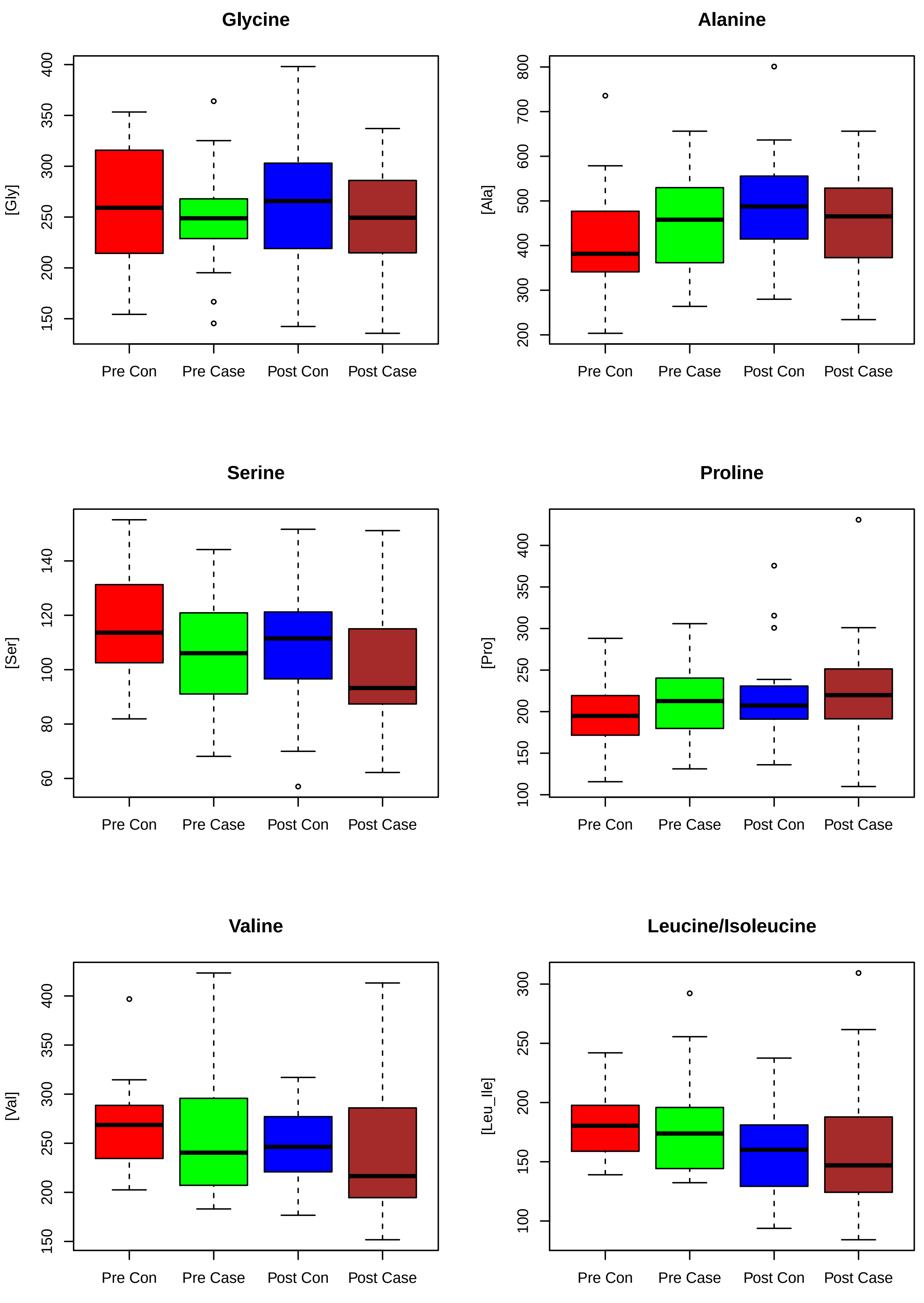

Glycine
[Gly]
150
200
250
300
350
400
Pre Con
Pre Case
Post Con
Post Case
Alanine
[Ala]
200
300
400
500
600
700
800
Serine
[Ser]
60
80
100
120
140
Proline
[Pro]
100
150
200
250
300
350
400
Valine
[Val]
150
200
250
300
350
400
Leucine/Isoleucine
[Leu_Ile]
100
150
200
250
300

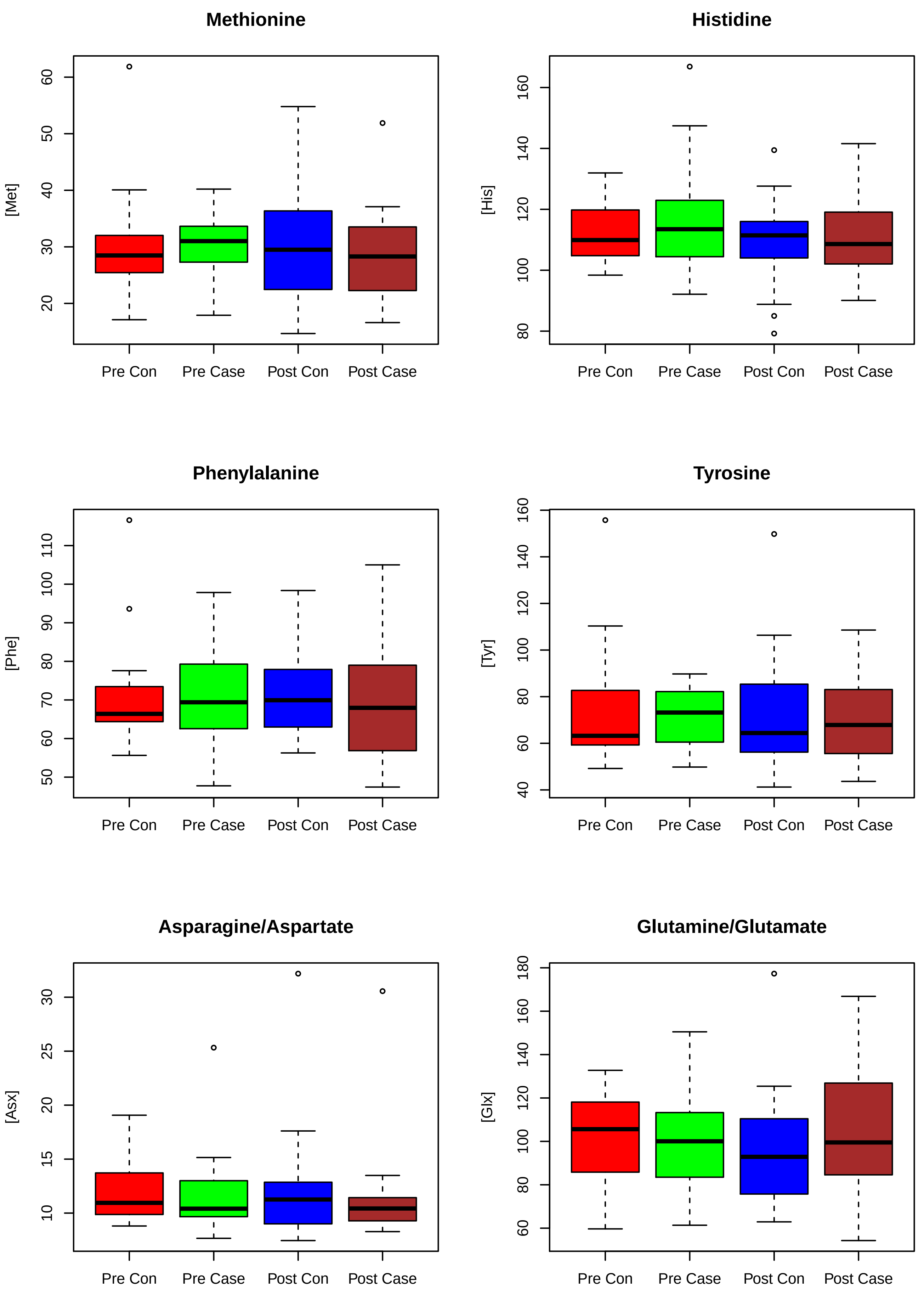
Methionine
[Met]
20 30 40 50 60
Pre Con
Pre Case
Post Con
Post Case
Histidine
[His]
80 100 120 140 160
Pre Con
Pre Case
Post Con
Post Case
Phenylalanine
[Phe]
50 60 70 80 90 100 110
Pre Con
Pre Case
Post Con
Post Case
Tyrosine
[Tyr]
40 60 80 100 120 140 160
Pre Con
Pre Case
Post Con
Post Case
Asparagine/Aspartate
[Asx]
10 15 20 25 30
Pre Con
Pre Case
Post Con
Post Case
Glutamine/Glutamate
[Glx]
60 80 100 120 140 160 180
Pre Con
Pre Case
Post Con
Post Case

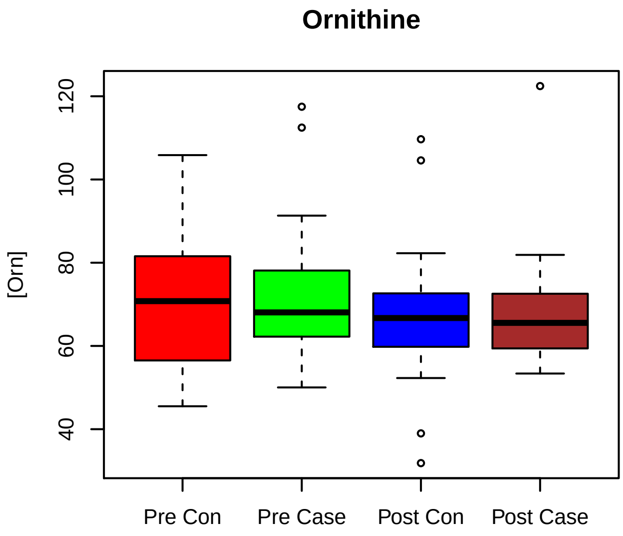
Ornithine
[Orn]
120
100
80
60
40
Pre Con
Pre Case
Post Con
Post Case

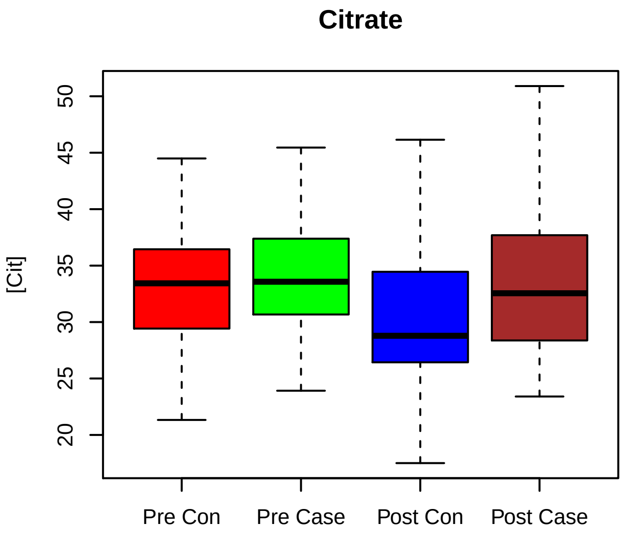
Citrate
[Cit]
50
45
40
35
30
25
20
Pre Con
Pre Case
Post Con
Post Case

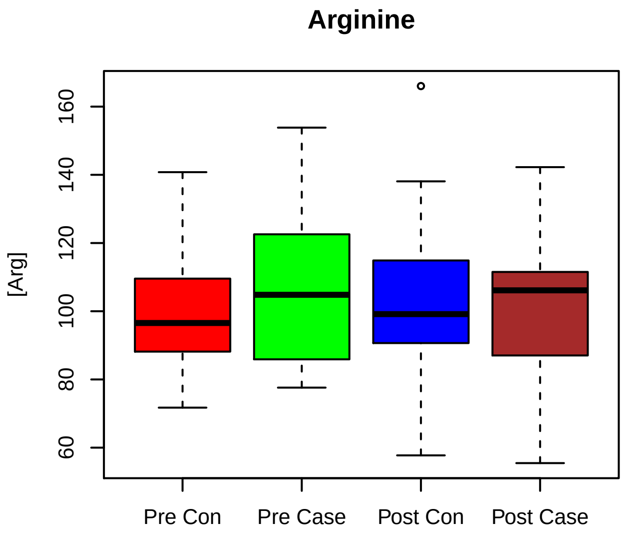
Arginine
[Arg]
160
140
120
100
80
60
Pre Con
Pre Case
Post Con
Post Case
